# The influence of breeding phenology on the genetic structure of four pond‐breeding salamanders

**DOI:** 10.1002/ece3.3060

**Published:** 2017-05-22

**Authors:** Jacob J. Burkhart, William E. Peterman, Emily R. Brocato, Kimberly M. Romine, M. Madeline S. Willis, Brittany H. Ousterhout, Thomas L. Anderson, Dana L. Drake, Freya E. Rowland, Raymond D. Semlitsch, Lori S. Eggert

**Affiliations:** ^1^ Division of Biological SciencesUniversity of MissouriColumbiaMOUSA; ^2^ School of Environment and Natural ResourcesThe Ohio State UniversityColumbusOHUSA; ^3^ Department of Biological SciencesUniversity of ArkansasFayettevilleARUSA; ^4^ Kansas Biological SurveyUniversity of KansasLawrenceKSUSA; ^5^ Department of Ecology and Evolutionary BiologyUniversity of ConnecticutStorrsCTUSA

**Keywords:** *Ambystoma*, amphibians, Caudata, landscape genetics, life history, *Notophthalmus viridescens*

## Abstract

Understanding metapopulation dynamics requires knowledge about local population dynamics and movement in both space and time. Most genetic metapopulation studies use one or two study species across the same landscape to infer population dynamics; however, using multiple co‐occurring species allows for testing of hypotheses related to different life history strategies. We used genetic data to study dispersal, as measured by gene flow, in three ambystomatid salamanders (*Ambystoma annulatum*,* A. maculatum*, and *A. opacum*) and the Central Newt (*Notophthalmus viridescens louisianensis*) on the same landscape in Missouri, USA. While all four salamander species are forest dependent organisms that require fishless ponds to reproduce, they differ in breeding phenology and spatial distribution on the landscape. We use these differences in life history and distribution to address the following questions: (1) Are there species‐level differences in the observed patterns of genetic diversity and genetic structure? and (2) Is dispersal influenced by landscape resistance? We detected two genetic clusters in *A. annulatum* and *A. opacum* on our landscape; both species breed in the fall and larvae overwinter in ponds. In contrast, no structure was evident in *A. maculatum* and *N. v. louisianensis*, species that breed during the spring. Tests for isolation by distance were significant for the three ambystomatids but not for *N. v. louisianensis*. Landscape resistance also contributed to genetic differentiation for all four species. Our results suggest species‐level differences in dispersal ability and breeding phenology are driving observed patterns of genetic differentiation. From an evolutionary standpoint, the observed differences in dispersal distances and genetic structure between fall breeding and spring breeding species may be a result of the trade‐off between larval period length and size at metamorphosis which in turn may influence the long‐term viability of the metapopulation. Thus, it is important to consider life history differences among closely related and ecologically similar species when making management decisions.

## 1 INTRODUCTION

A primary goal in landscape genetic studies is to understand the degree to which dispersal, as measured by gene flow, is facilitated or impeded by environmental factors (Manel & Holderegger, 2013; Wagner & Fortin, 2013). Dispersal, the permanent movement of an individual away from its natal location, is influenced by both intrinsic (e.g., morphology, physiology, behavior, life history) and extrinsic factors (e.g., density dependence, habitat quality), and is the primary mechanism for maintaining gene flow among populations (Clobert, Le Galliard, Cote, Meylan, & Massot, 2009; Einum, Sundt‐Hansen, & Nislow, 2006). Successful dispersal is sometimes aided by morphological adaptations, such as the winged versus nonwinged morphologies of crickets (Simmons & Thomas, 2004), or behavioral adaptations, such as the propensity of cane toads to move faster and make more directed movements at the range front (Phillips, Brown, Travis, & Shine, 2008). Although these mechanisms are important drivers of dispersal ability, successful dispersal also relies on the timing and duration of dispersal events.

The majority of landscape genetic studies, regardless of taxa, have focused on one or a few species (Cushman, Landguth, & Flather, 2013; Dyer, Nason, & Garrick, 2010; Greenwald, Purrenhage, & Savage, 2009; Kierepka & Latch, 2016; Mims, Phillipsen, Lytle, Hartfield Kirk, & Olden, 2015; Peterman et al., 2015; Schwartz et al., 2009; Whiteley, McGarigal, & Schwartz, 2014). Although single species studies help address interesting questions, community structure can be better understood by investigating how the landscape influences population dynamics of multiple species and how those species interact (Manel & Holderegger, 2013). Additionally, using multiple species that differ in life history traits on the same landscape allows for testing of hypotheses with regard to the effect of life history on genetic differentiation (Mims et al., 2015; Whiteley, Spruell, & Allendorf, 2004).

Life history traits, such as timing of breeding, oviposition strategies, growth rates, and fecundity, can all affect a species’ ability to survive and successfully disperse to new habitat patches. Reduced dispersal between suitable habitat patches in turn decreases gene flow among habitat patches, which results in greater genetic differentiation. Across taxa, observed differences in genetic differentiation among closely related, co‐occurring species can often be attributed to differences in life history traits (Dawson, Louie, Barlow, Jacobs, & Swift, 2002; Kierepka, Anderson, Swihart, & Rhodes, 2016; Whiteley et al., 2004). In fragmented landscapes, habitat specialist species may exhibit higher degrees of genetic isolation than generalist species as is the case with eastern chipmunks (*Tamias striatus*) and white‐footed mice (*Peromyscus leucopus*) in Indiana (Kierepka et al., 2016). Whiteley et al. (2004) found evidence that differences in population size and spawning habitat specificity can lead to higher levels of philopatry in bull trout (*Salvelinus confluentus*) which results in higher levels of genetic differentiation than the generalist mountain whitefish (*Prosopium williamsoni*) within the same river network. Similarly, differences in fecundity, larval period length, and population size were hypothesized to drive differential phylogeographic structure in two sympatric marine gobies (Dawson et al., 2002). Therefore, when estimating gene flow for species with complex life cycles (i.e., species that undergo an abrupt change in ontogeny, physiology, and behavior; Wilbur, 1980), it is important to consider the contribution of differences in life history traits.

Organisms with complex life cycles, such as pond‐breeding amphibians, parasites, aquatic invertebrates, and butterflies, require breeding habitat that is present for the duration of their larval period to successfully metamorphose without catastrophic reproductive failure (Taylor, Scott, & Gibbons, 2006; Wilbur, 1980). Without successful metamorphosis, there is no recruitment of individuals and thus no contribution of genes to the breeding population, dispersal to new breeding populations, or colonization of new populations (Petranka, 2007; Semlitsch, Scott, Pechmann, & Gibbons, 1996; Werner, Relyea, Yurewicz, Skelly, & Davis, 2009). Furthermore, the timing of metamorphosis may greatly influence the ability of an individual to disperse. For organisms such as amphibians that are prone to desiccation, individuals that metamorphose later in the summer may encounter a more inhospitable habitat matrix than those that metamorphose earlier in the year when moisture is higher due to spring rains. Additionally, animals that metamorphose at smaller sizes have a higher surface area to volume ratio and are more prone to desiccation which can further reduce the dispersal ability of the organism, especially when encountering an inhospitable landscape (Grover & Ross, 2000; Peterman & Semlitsch, 2014).

**Figure 1:**
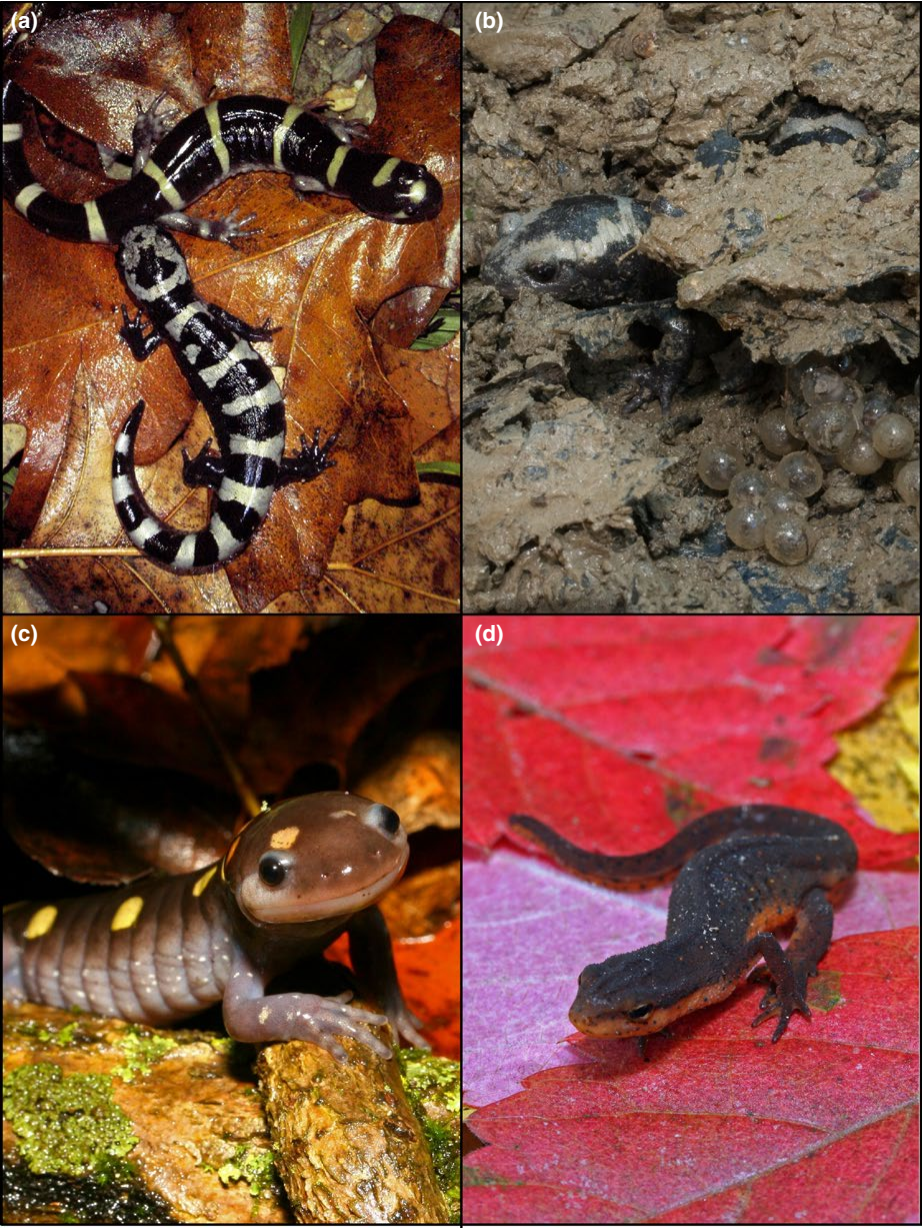
(a) Male *Ambystoma annulatum* (ringed salamander; top) and male *Ambystoma opacum* (marbled salamander*;* bottom) during the annual fall migration to breeding ponds. (b) Female *A. opacum* guarding her nest of eggs on a pond margin. (c) Adult *A. maculatum* (spotted salamander) and (d) *Notophthalmus viridescens louisianensis* (central newt) making their spring breeding migrations. Photographs by D. L. Drake (a), W. E. Peterman (b–d)

In this study, we investigated patterns of genetic structure and diversity for four salamander species that co‐occur on the same landscape in Missouri. The ringed salamander (*Ambystoma annulatum*; Figure 1a) is an Ozark Highland and Ouachita Mountain endemic, whereas the marbled salamander (*A. opacum*; Figure 1b), the spotted salamander (*A. maculatum*; Figure 1c), and the central newt (*Notophthalmus viridescens louisianensis*; Figure 1d) are distributed throughout much of the eastern United States with *A. maculatum* and *N. v. louisianensis* being found as far north as southeastern Canada (Petranka, 1998). *Ambystoma annulatum* and *A. opacum* are fall breeding species whose larvae overwinter in the ponds and metamorphose in April–June (Hocking et al., 2008; Semlitsch, Anderson, Osbourn, & Ousterhout, 2014). Both species breed in late August–November and female *A. annulatum* oviposit eggs on submerged substrates, whereas *A. opacum* oviposit on the margin of the wetlands in shallow depressions and eggs hatch upon inundation (Hocking et al., 2008). Adult *A. maculatum* make an annual breeding migration in February–March, females oviposit on submerged substrates, and larvae metamorphose in late June–August with some metamorphosing as late as October (Hocking et al., 2008; Semlitsch & Anderson, 2016). Adult *N. v. louisianensis* migrate to ponds in February–March, females lay eggs singly over a period of days to weeks, adults remain present in ponds throughout the summer, and juveniles metamorphose in August–October (Gill, 1978; Hocking et al., 2008). In all four species, juveniles emigrate from ponds during or immediately after pulses of rain (Pittman, Osbourn, & Semlitsch, 2014) to forested habitats where they are highly fossorial, residing in small mammal burrows (Petranka, 1998; Semlitsch, 1981).

Although these four species are ecologically similar in that they are all forest dependent and breed in fishless ponds, differential interactions with landscape features can exist among co‐occurring taxa on the same landscape (Goldberg & Waits, 2010; Mims et al., 2015; Peterman et al., 2015; Whiteley et al., 2014). Mims et al. (2015) found that water dependency was the primary driver of genetic diversity of three anuran species in the Madrean Sky Islands of Arizona. The anuran species with longer larval periods were dependent on more permanent sources of water for breeding and larval survival, and this led to decreased metapopulation connectivity as these patches were more limited (Mims et al., 2015). Similarly, comparative studies of two ambystomatid salamanders by Peterman et al. (2015) and Whiteley et al. (2014) observed that fall breeding species (*A. annulatum* and *A. opacum*, respectively) have more limited gene flow across the same spatial scale than spring breeding species (*A. maculatum*). The authors posited that differences in breeding phenology and the presence of suitable water sources on the landscape during breeding events shape genetic structure as spring breeding species can utilize a wider variety of breeding habitats (e.g., tire ruts, shallow depressions) and as a result are more adept dispersers (Peterman et al., 2015; Whiteley et al., 2014).

Our study investigates whether the genetic structure and diversity of four co‐occurring salamander species is influenced by breeding phenology. We test this by extending the work of Peterman et al. (2015) by collecting additional genetic data for *A. annulatum* and *A. maculatum* as well as adding genetic data for a replicate fall breeding (*A. opacum*) and spring breeding (*N. v. louisianensis*) salamander species. We first assessed the patterns of genetic diversity and genetic structure for all four species across the same study landscape. We then tested the effect of the interpond landscape matrix on genetic differentiation using a resistance modeling approach.

## 2 MATERIALS AND METHODS

### 2.1 Study area and sample collection

**Figure 2:**
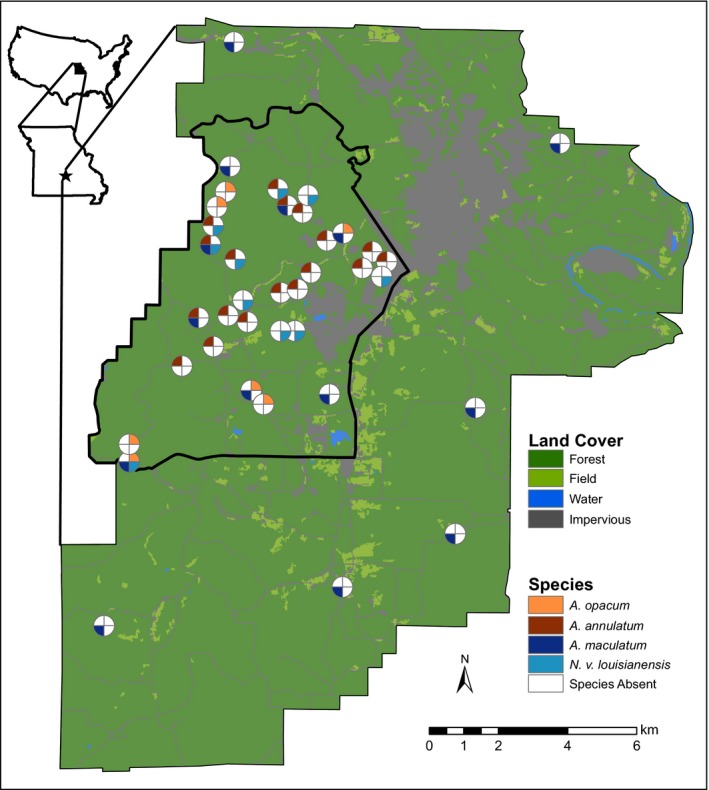
Sampling locations at Fort Leonard Wood in Pulaski County, Missouri. Sections of each pie chart represent presence (colors) or absence (white) of genetic samples for each species. Although multiple species may occupy ponds, this study includes only those ponds from which a sufficient number of samples were genotyped. Background is the reclassified 30 × 30 m land use, land cover surface and the thick black border indicates the focal study area

We conducted our study at Fort Leonard Wood (FLW) military training facility in Pulaski County, Missouri, USA (Figure 2). This 24,686 ha facility is actively used year round by all five branches of the armed forces for training and is approximately 80% forested, primarily oak‐hickory forest (*Quercus* spp. and *Carya* spp. canopy; *Rhus aromatic* and *Cornus florida* understory) with intermixed short‐leaf pine plantations (*Pinus echinata*). There are approximately 500 constructed or unintentional bodies of water (e.g., tire ruts) on the FLW landscape with varying hydroperiods and aquatic communities; however, the majority are small (<0.04 ha), fishless, manmade ponds (Peterman, Anderson, Drake, Ousterhout, & Semlitsch, 2014).

We collected *A. maculatum* samples from ponds of varying hydroperiod across the entire landscape, whereas we collected *A. annulatum*,* A. opacum*, and *N. v. louisianensis* from ponds over a 7,140‐ha subset of FLW (focal area; Figure 2). Tissue samples were collected from all four species between spring 2013 and summer 2014 (Table 1). We collected all samples during the same breeding season to minimize the among‐year variation in breeding effort as most female ambystomatids do not breed annually (Semlitsch et al., 1996; Titus, Madison, & Green, 2014). When ponds were located within 100 m of each other, we pooled all individuals from those ponds into one group for downstream analyses. We chose the 100‐m cutoff because 95% of adult salamanders are assumed to use the terrestrial landscape within 300 m from the edge of their breeding pond (Rittenhouse & Semlitsch, 2007) and ambystomatid adults have been observed utilizing a different breeding pond within 100 m when an annual breeding pond is dry (Gamble, McGarigal, & Compton, 2007; Trenham, Koenig, & Shaffer, 2001).

**Table 1:** Salamander samples collected and analyzed for this study

Species	Collection period	*N* ponds	*N* samples retained	*N* samples collected	Sample stage
*Ambystoma annulatum*	Fall 2013	26	421	488	Late stage embryos and larval tail clips
*Ambystoma maculatum*	Spring 2013	17	332	342	Late stage embryos
*Ambystoma opacum*	Fall 2013	8	129	133	Larval tail clips
*Notophthalmus viridescens louisianensis*	Summer 2014	11	110	110	Adult tail clips

### 2.2 Genetic analyses

We extracted DNA from tissue samples using Instagene (Bio‐Rad, Hercules, CA, USA) following the protocol outlined in Peterman, Connette, Spatola, Eggert, and Semlitsch (2012). We genotyped *A. annulatum* at 19 microsatellite loci (Table S1; Peterman, Pauley et al., 2013), *A. opacum* at 13 microsatellite loci (Table S2; Martin, 2013; Nunziata, Scott, Jones, Hagen, & Lance, 2011; Peterman, Pauley et al., 2013), *A. maculatum* at 18 microsatellite loci (Table S3; Peterman, Brocato et al., 2013), and *N. v. louisianensis* at nine microsatellite loci (Table S4; Croshaw & Glenn, 2003; Jones, Blouin, & Arnold, 2001; May, 2011). All forward primers were fluorescently labeled and amplified following the polymerase chain reaction (PCR) protocol of Peterman, Brocato et al. (2013). With the exception of *N. v. louisianensis*, for which genotyping was performed in single‐locus reactions, we arranged loci into two multiplexes as described in Peterman, Brocato et al. (2013), Peterman, Pauley et al. (2013), or Table S2 (*A. opacum*). We sized PCR products on an ABI 3730xl DNA Analyzer (Applied Biosystems, Foster City, CA, USA) using Liz 600 size standard at the University of Missouri DNA Core Facility and scored genotypes using GeneMarker v.1.97 (Softgenetics, State College, PA, USA). Samples that amplified at <80% of loci were removed from the dataset. We tested for the presence of full siblings with COLONY v2.0.5.9 (Jones & Wang, 2010). We set male and female mating to polygamous without inbreeding and ran the analysis as a long run with full likelihood, high precision, and no sibship prior. If pairs of samples from the same population were identified as having a >95% posterior probability likelihood of being related at the level of parent‐offspring or full‐sibling, we haphazardly selected one of the samples to keep for downstream analysis.

We tested for deviations from expected heterozygosity values under Hardy–Weinberg Equilibrium (HWE) and linkage disequlibrium among pairs of loci with Genepop on the Web (Raymond & Rousset, 1995; Rousset, 2008). We conducted both tests using 1,000 dememorization steps and 100 batches with 1,000 interations per batch and assessed the significance of our results following a Bonferroni correction for the number of comparisons (Rice, 1989). We used the “PopGenReport” package (Adamack & Gruber, 2014) in R (R Core Team 2016) to test for the presence of null alleles.

We did not correct for sample sizes across species because the metrics we used for genetic diversity (rarefied allelic richness) and differentiation (*F*’_ST_) account for differences in sample sizes. We calculated rarefied allelic richness in the program HP‐Rare (Kalinowski, 2005). Then, we used GenoDive v2.0 (Meirmans & Van Tienderen, 2004) to calculate observed and expected heterozygosity, the standardized fixation index *F*’_ST_, and inbreeding coefficients (*F*_IS_). We tested for isolation by distance (IBD) using the “vegan” package (Oksanen et al., 2016) for R (R Core Team, 2016) and tested for significant differences in the slopes between species with an ANCOVA in R. We assessed population genetic clustering using Bayesian assignment methods implemented in STRUCTURE v2.3.4 (Pritchard, Stephens, & Donnelly, 2000) and BAPS v6.0 (Corander & Marttinen, 2006). In STRUCTURE, we used 100,000 burn‐in steps followed by 500,000 MCMC iterations for *K* = 2–15 under an admixture model with correlated allele frequencies and no location prior. To evaluate the STRUCTURE results, we calculated the rate of change in the log likelihood between *K* values (∆*K*; Evanno, Regnaut, & Goudet, 2005) in Structure Harvester (Earl & vonHoldt, 2012). In BAPS, we determined the most likely number of genetic partitions per species with a two‐step process. First, we used the spatial clustering of individuals option to assign each sample to its most likely genetic partition. Then, we then ran an admixture analysis to refine our results (Corander & Marttinen, 2006). The admixture approach uses spatial information, Voroni tessellation, and Markov Random fields to determine the maximum number of population clusters (*K*). As in STRUCTURE, we tested for 2–15 potential clusters using ten replicates for each potential cluster number within each species. In both STRUCTURE and BAPS, we tested for hierarchical substructure within each putative cluster in a separate analysis for *K* = 2–15 with the same criteria used in the initial analyses.

### 2.3 Landscape resistance analyses

We generated landscape resistance surfaces using ArcGIS v10.3 (ESRI, Redlands, CA, USA) to test our hypothesis that the species differ in their response to the landscape. We obtained our land cover data (30 × 30 m resolution) and digital elevation model (DEM; 90 × 90 m resolution) from the U.S. Geological Survey National Map server (USGS, http://view.nationalmap.gov). All other resistance surfaces were derived from 90‐m resolution USGS DEM layer in ArcGIS (ESRI) following the methods outlined in Peterman and Semlitsch (2013). Because the resolution of land cover data was 30 m, we resampled this surface to 90‐m resolution to match our DEM‐derived resistance surfaces. Resistance surfaces were defined as follows: eastness (sine of aspect; values range from 1 = east to −1 = west), northness (cosine of aspect; values range from 1 = north to −1 = south), streams (binary), percent slope, topographic position index (TPI; Jenness, 2006), topographic wetness index (TWI; Theobold, 2007), and distance from ravines. We used the slope position classification for our TPI resistance surface where the landscape was classified as follows: 1—hilltop, 2—upper slope, 3—mid‐slope, 4—flat, 5—lower slope, 6—valley bottom using a 270 × 270 m sliding window. Topographic wetness index is a measure that is used to estimate the influence of topography on hydrological processes. None of our resistance surfaces were strongly correlated with each other as Pearson's correlation coefficients never exceeded *r* = .70.

We used the “ResistanceGA” package (Peterman, 2014) in R to assess the effects of distance and landscape resistance on pairwise genetic differentiation. ResistanceGA uses a genetic algorithm (GA; Scrucca, 2013) to adaptively optimize resistance surfaces through a series of transformations (continuous resistance surfaces) or by assignment of resistance values (categorical resistance surfaces). At each iteration, the relative support for a landscape resistance surface was assessed using linear mixed‐effects models fit with “lme4” (Bates, Mächler, Bolker, & Walker, 2014) using a maximum‐likelihood population effects (MLPE) parameterization to account for the nonindependence of values within pairwise distance matrices (Clarke, Rothery, & Raybould, 2002; van Strien, Keller, & Holderegger, 2012). Pairwise *F*’_ST_ was used as the dependent variable and scaled and centered circuit resistance distance between populations was the independent variable. Model fits were assessed using AICc calculated from the linear mixed‐effects models. Optimization proceeded until no further improvement of AICc could be achieved (for detailed description of ResistanceGA see Peterman, 2014). For species where more than one model emerged as describing genetic structure better than distance alone, we created a composite resistance surface for each species using a combination of all resistance surfaces that performed better than distance alone. We optimized the composite resistance surfaces using the same methods we employed for single resistance surfaces and compared the performance of the composite resistance surface and single resistance surfaces using AIC_C_.

## 3 RESULTS

### 3.1 Genetic analyses

We successfully genotyped 472 of 488 *A. annulatum*, 133 of 133 *A. opacum*, 340 of 340 *A. maculatum*, and 110 of 110 *N. v. louisianensis* at >80% of all loci. We observed a low frequency of full siblings in each of our datasets; 3.0% of the 472 *A. annulatum*, 9.8% of the 133 *A. opacum*, 0.0% of the 340 *A. maculatum*, and 1.8% of the 110 *N. v. louisianensis* samples. After removing full‐siblings and individuals with incomplete genotypes, we further refined our dataset by eliminating all ponds or pond groupings (ponds within 100 m of each other) from which we did not have at least nine fully genotyped individuals.

When testing for independence of loci in our dataset, no population or locus significantly deviated from HWE following Bonferroni's corrections for *A. annulatum*,* A. maculatum*, or *N. v. louisianensis*. However, for *A. opacum* two loci (Aa‐29 and Aa‐312) deviated significantly from expectations under HWE due to heterozygote deficiency and were removed from further analyses. We did not observe evidence of null alleles at any loci or of linkage disequilibrium among pairs of loci in any species. Our final dataset included 421 *A. annulatum* from 26 sites at 19 loci, 123 *A. opacum* from eight sites at 11 loci, 332 *A. maculatum* from 17 sites at 18 loci, and 108 *N. v. louisianensis* from 11 sites at nine loci.

Across all ponds, the observed heterozygosity for *A. annulatum* was 0.682 (Table 2), *A. opacum* was 0.440 (Table 2), *A. maculatum* was 0.572 (Table 2), and *N. v. louisianensis* was 0.537 (Table 2). The estimated level of inbreeding, *F*_IS_, was 0.011 for *A. annulatum* (Table 2), 0.231 for *A. opacum* (Table 2), 0.029 for *A. maculatum* (Table 2), and 0.271 for *N. v. louisianensis* (Table 2). Average pairwise *F*’_ST_ was 0.151 for *A. annulatum* (Table 2 and Table S5), 0.052 for *A. opacum* (Table 2 and Table S6), 0.065 for *A. maculatum* (Table 2 and Table S7), and −0.050 for *N. v. louisianensis* (Table 2 and Table S8). The rarefied allelic richness, A_R_, did not differ significantly across species. *Ambystoma annulatum* averaged 3.57 alleles per locus, *A. opacum* averaged 2.99 alleles per locus, *A. maculatum* averaged 3.09 alleles per locus, and *N. v. louisianensis* averaged 4.01 alleles per locus.

**Table 2:** Summary of genetic diversity for each species in our study

Species	*A*	*A*_R_	*H*_O_	*H*_E_	*F*_IS_	*F*’_ST_
*A. annulatum*	8.16 ± 0.88	3.57 ± 0.39	0.682 ± 0.031	0.690 ± 0.031	0.011 ± 0.042	0.151 ± 0.047
*A. opacum*	8.81 ± 1.41	2.99 ± 0.32	0.440 ± 0.075	0.601 ± 0.082	0.231 ± 0.086	0.052 ± 0.043
*A. maculatum*	7.83 ± 0.89	3.09 ± 0.21	0.572 ± 0.047	0.606 ± 0.049	0.029 ± 0.030	0.065 ± 0.030
*N. v. louisianensis*	10.56 ± 1.94	4.01 ± 0.38	0.537 ± 0.084	0.739 ± 0.052	0.271 ± 0.105	−0.050 ± 0.092

Values reported are mean ± standard deviation.

*A* = number of alleles; *A*_R_ = rarified allelic richness; *H*_O_ = observed heterozygosity; *H*_E_ = expected heterozygosity; *F*_IS_ = inbreeding coefficient; *F*′_ST_ = standardized measure of population differentiation (Meirmans, 2006).

**Figure 3:**
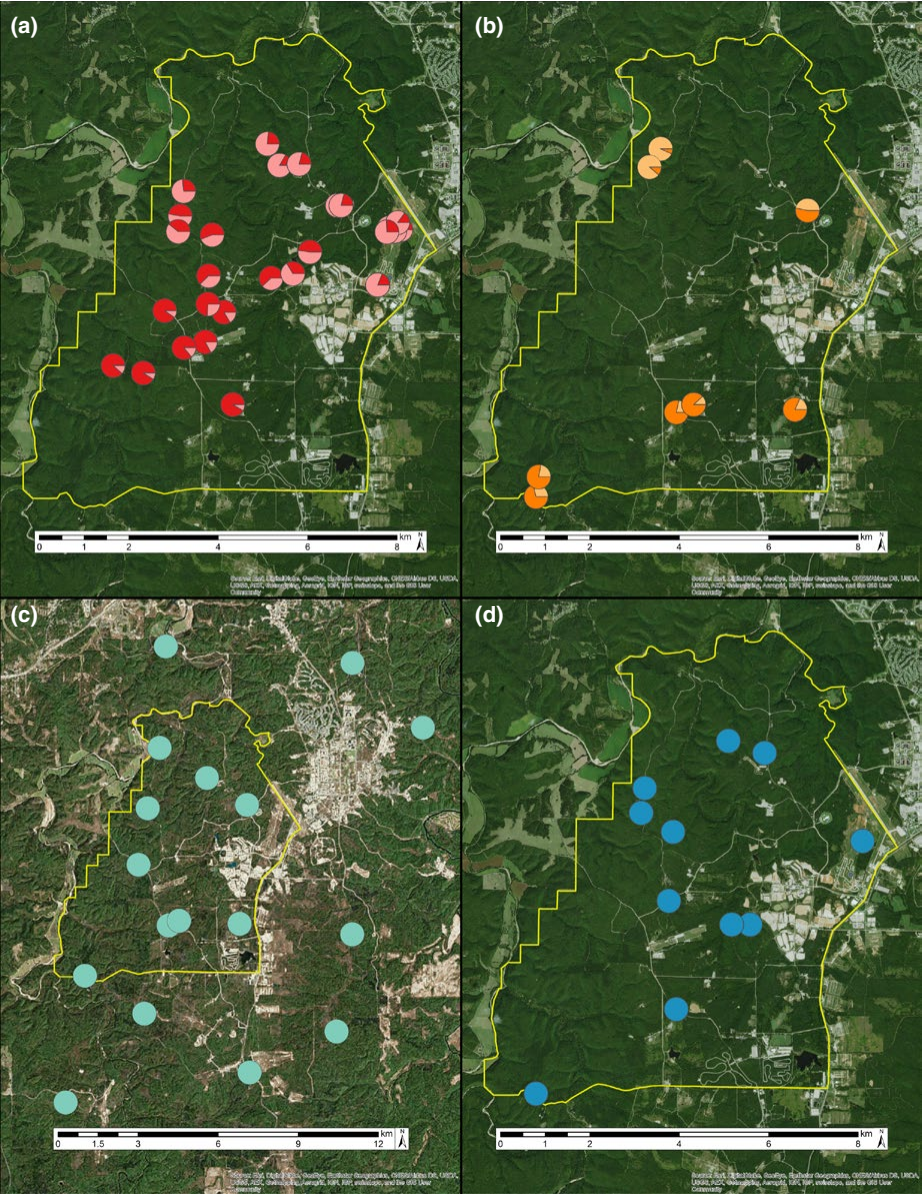
Spatial arrangement of genetic clusters for four salamander species at Fort Leonard Wood, MO: (a) *Ambystoma annulatum*, (b) *A. opacum*, (c) *A. maculatum*, and (d) *Notophthalmus viridescens louisianensis*. Each pie chart signifies the location of a sample pond and different color shades in panels (a) and (b) correspond to the proportion of each pond's genotype that assigns to each putative genetic cluster. The yellow line indicates the boundary of the focal area, and background is satellite imagery (from Google Earth)

STRUCTURE analyses detected genetic clustering within two of the four species; both fall breeding species, *A. annulatum* and *A. opacum*, group as two genetic clusters on our landscape (Δ*K* = 134.35 and Δ*K* = 50.00, respectively; Figure 3a,b). The results of clustering tests in BAPS were broadly concordant with those from STRUCTURE (Fig. S1). In both STRUCTURE and BAPS, we found no evidence for substructure within each of these clusters. *Ambystoma annulatum* has one cluster in the northeast and one cluster in the southwest with an admixture zone running diagonally through the central portion of the focal area (Figure 3a), whereas *A. opacum* has a cluster in the northwest and a cluster in the southeast with a possible admixture zone between these regions (Figure 3b). The two spring breeding species, *A. maculatum* and *N. v. louisianensis*, each formed a single genetic cluster (Figure 3c,d).

### 3.2 Landscape analyses

**Figure 4:**
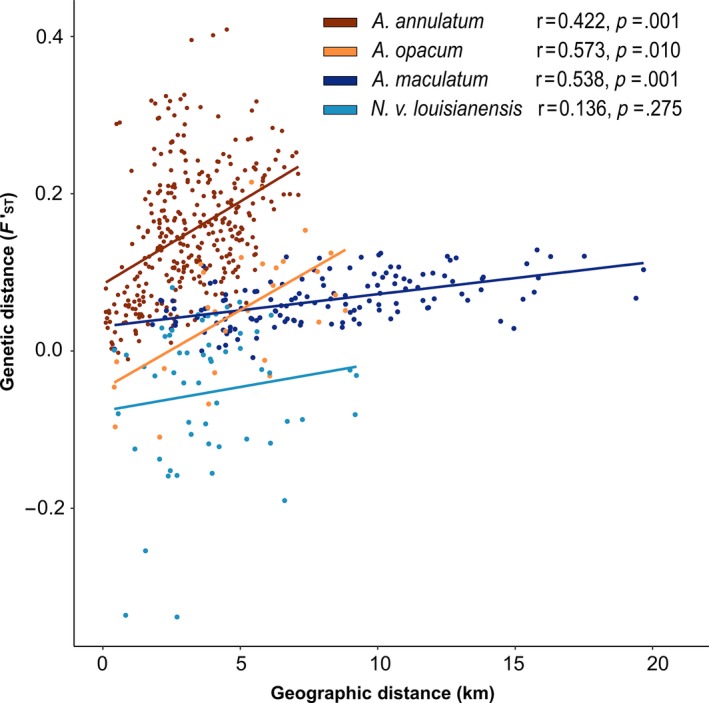
Isolation by distance plots for four species of salamanders at Fort Leonard Wood, MO. Each point corresponds to observed pairwise geographic and genetic distances between two ponds and lines correspond to the predicted relationship between genetic and geographic distance

Tests for IBD were significant in all three ambystomatid species but not in *N. v. louisianensis* (Figure 4). There were significant differences in the relationship between genetic distance and geographic distance among species (*F*_7, 536_ = 14.33, *p* < .001). The slopes of genetic distance and geographic distance for both *A. annulatum* (β = .021, 95% CI = 0.016–0.260) and *A. opacum* (β = .020, 95% CI = 0.008–0.032) were not significantly different from each other but both species have significantly greater slopes than *A. maculatum* (β = .004, 95% CI = 0.003–0.005) and *N. v. louisianensis* (β = 0.006, 95% CI = −0.006 to 0.018). In addition, we observed strong support in *A. annulatum* and marginal support in the other three species for isolation by resistance being a better predictor of genetic structure than distance alone (Table 3). In our single surface resistance optimization for *A. annulatum*, genetic distance was best predicted by TPI ($R_{m}^{2}$ = .39; discrete transformation: hilltop = 1, upper slope = 10.79, mid‐slope = 27.43, flat = 19.05, lower slope = 296.03, valley bottom = 331.01) with distance from ravines also being strongly supported ($R_{m}^{2}$ = .54; Table 3). Distance from ravine was optimized such that areas closer to the ravines had high resistance, and resistance decreased as distance from the ravine increased (Fig. S2). The composite resistance surface, which combined TPI and distance from ravines, was our top model in which hilltops and upper slopes have lower resistance than lower slopes and valley bottoms ($R_{m}^{2}$ = .63). *Ambystoma opacum* genetic differentiation was best predicted by the eastness resistance surface ($R_{m}^{2}$ = .30); moreover, TWI, distance from ravine, and percent slope resistance surfaces all had ΔAIC_C_ < 2.00 (Table 3 and Fig. S3). Using ΔAIC_C,_ the composite resistance surface was not strongly supported for this species although it described approximately 1.5 times the variance of the eastness surface alone ($R_{m}^{2}$ = .50). For *A. maculatum*, our best supported model was northness ($R_{m}^{2}$ = .43) with eastness ($R_{m}^{2}$ = .42) and percent slope ($R_{m}^{2}$ = .46) both having ΔAIC_C_ < 2.00 (Table 3; Fig. S4). Our composite surface, which combined northness, eastness, slope, streams, TWI, and distance from ravines, was well supported (ΔAIC_C_ = 0.40; $R_{m}^{2}$ = .50), although it was not identified as the top model (Table 3). For *N. v. louisianensis*, eastness was our top model and all other surfaces had ΔAIC_C_ > 4.00 and $R_{m}^{2}$ < .30 (Table 3; Fig. S5).

**Table 3:** Landscape genetic model rankings for the different resistance surfaces tested across all four species

Surface	Type	Transformation	Shape	Max	AIC_C_	ΔAIC_C_	$R_{m}^{2}$	$R_{c}^{2}$
*A. annulatum*								
Composite					**−1,264.60**	**0.00**	**.63**	**.82**
TPI	Categorical	–	–	–	−**1,254.47**	**10.13**	**.39**	**.77**
Ravine	Continuous	Inverse monomolecular	**0.07**	**30.92**	−**1,253.52**	**11.09**	**.54**	**.79**
TWI	Continuous	Inverse Ricker	2.16	132.73	−1,237.27	27.33	.49	.86
Slope	Continuous	Reverse Ricker	1.42	437.57	−1,235.37	29.24	.38	.82
Streams	Categorical	–	–	–	−1,231.28	33.33	.30	.76
Eastness	Continuous	Inv.‐Rev. monomolecular	2.85	31.82	−1,226.73	37.87	.48	.84
Northness	Continuous	Reverse Ricker	3.22	456.64	−1,224.56	40.04	.41	.79
Distance	Uniform	–	–	–	−1,216.68	47.92	.27	.77
*A. opacum*								
Eastness	Continuous	Inv.‐Rev. Monomolecular	**1.63**	**39.40**	−**59.97**	**0.00**	**.30**	**.40**
TWI	Continuous	Inverse Ricker	**2.79**	**499.11**	−**59.21**	**0.76**	**.24**	**.38**
Ravine	Continuous	Inv.‐Rev. monomolecular	**1.16**	**449.26**	−**59.01**	**0.96**	**.23**	**.39**
Slope	Continuous	Ricker	**0.53**	**201.43**	−**58.61**	**1.36**	**.22**	**.44**
Northness	Continuous	Monomolecular	0.47	439.08	−57.22	2.75	.11	.35
Distance	Uniform	–	–	–	−56.88	3.08	.04	.35
Streams	Categorical	–	–	–	−56.63	3.33	.05	.35
TPI	Categorical	–	–	–	−55.36	4.61	.29	.45
Composite					1.32	10.86	.50	.68
*A. maculatum*								
Northness	Continuous	Reverse monomolecular	**0.34**	**54.45**	−**638.83**	**0.00**	**.43**	**.55**
Eastness	Continuous	Reverse Ricker	**0.59**	**6.49**	−**638.43**	**0.39**	**.42**	**.55**
Composite					−**638.43**	**0.40**	**.50**	**.57**
Slope	Continuous	Reverse Ricker	**0.73**	376.50	−**638.24**	**0.59**	**.46**	**.58**
Streams	Categorical	–	–	–	−636.79	2.04	.42	.56
TWI	Continuous	Reverse Ricker	0.65	383.86	−636.65	2.17	.42	.56
Ravine	Continuous	Revere monomolecular	12.42	1.88	−636.35	2.48	.42	.56
Distance	Uniform	–	–	–	−635.65	3.17	.39	.55
TPI	Categorical	–	–	–	−627.97	10.86	.50	.68
*N. v. louisianensis*								
Eastness	Continuous	Ricker	**1.29**	**198.64**	−**138.31**	**0.00**	**.30**	**.60**
Ravine	Continuous	Ricker	1.75	202.84	−136.29	2.01	.19	.56
TPI	Categorical	–	–	–	−135.97	2.34	.27	.58
Northness	Continuous	Ricker	1.39	112.39	−135.96	2.34	.15	.56
Slope	Continuous	Inverse Ricker	0.68	224.02	−135.78	2.53	.15	.55
TWI	Continuous	Inv.‐Rev monomolecular	2.37	489.60	−135.42	2.89	.07	.52
Distance	Uniform	–	–	–	−134.66	3.64	.03	.54
Streams	Categorical	–	–	–	−134.48	3.83	.03	.54

Bolded values indicate models with ΔAIC_C_ < 2.0 in either the single surface or multiple surface optimizations.

Composite = a combined resistance surface for all surfaces with ΔAIC_C_ < 4.0; Transformation = best performing transformation of continuous resistance values selected by ResistanceGA; shape = optimal value for the shape parameter for the transformation; max = maximum value for the transformation of resistance values; $R_{m}^{2}$ = marginal *R* ^2^ value; $R_{c}^{2}$ = conditional *R* ^2^ value; TPI = topographic position index; TWI = topographic wetness index; *K* = number of parameters in model.

## 4 DISCUSSION

We observed different patterns of genetic diversity and structure among four salamander species that co‐occur on the landscape. Specifically, *A. annulatum* and *A. opacum* each consist of two genetic clusters on the landscape, whereas *A. maculatum* and *N. v. louisianensis* each consist of a single genetic cluster. Additionally, average pairwise *F*’_ST_ values were higher for *A. annulatum* than for *A. opacum*,* A. maculatum*, and *N. v. louisianensis*. Given that these values were measured over distances of <8 km for *A. annulatum* and *A. opacum*, and <20 km for *A. maculatum*, the observed values indicate strong genetic differentiation present across our study area.

At the scale of our study, we observed a strong and significant signal of IBD in three of the four study species (*A. annulatum*,* A. opacum*, and *A. maculatum*) and these significant relationships were observed over a smaller spatial scale for *A. annulatum* and *A. opacum* than for *A. maculatum* (Figure 4). This suggests that *A. annulatum* and *A. opacum* are more dispersal limited than *A. maculatum* on the same landscape; a phenomenon supported by previous studies that investigated the genetic and demographic dispersal of our study species. In a 7‐year metapopulation study of *A. opacum* in Massachusetts, Gamble et al. (2007) found that the maximum demographic dispersal distance of juvenile *A. opacum* was 1,300 m. The average genetic dispersal distance for *A. annulatum* has been estimated to be 1,693 m which is significantly less than the estimated 2,050 m genetic dispersal distance for *A. maculatum* (Peterman et al., 2015). Observed demographic dispersal distances are lacking for both *A. annulatum* and *A. maculatum*; however, adult *A. maculatum* have been observed moving 756 m during postbreeding emigrations (Madison, 1997) and single night total distance movements of 20–50 m for *A. annulatum* (Osbourn, 2012) and 53.44 m have been documented for *A. maculatum* (Pittman & Semlitsch, 2013). Furthermore, laboratory‐based movement assays of *A. annulatum* and *A. maculatum* found that *A. annulatum* have a greater maximum movement distance but smaller median dispersal distance than *A. maculatum* suggesting that *A. annulatum* are capable of moving farther but do so less often than *A. maculatum* (B. Ousterhout, *unpublished data*). Similarly, the lack of an IBD effect in *N. v. louisianensis* is not surprising as this species has been found to be capable of dispersing at least 3 km (Gill, 1978, 1979). Thus, the lack of spatial genetic structure and the lower degree of genetic differentiation on our study landscape for *A. maculatum* and *N. v. louisianensis* are likely a result of the ability and or propensity of these organisms to disperse over greater distances than either *A. annulatum* or *A. opacum*.

Across all species, we found support for landscape resistance describing genetic differentiation better than distance alone. With *A. annulatum*, we observed the strongest support for our composite resistance surface that combined TPI and distance from ravines. For this, surface ridges, flat areas, and upper slopes had lower resistance values than mid‐slopes, lower slopes, and valley bottoms. As ridges and higher slopes in the Missouri Ozarks tend to be warmer and drier than lower slopes and valley bottoms (Peterman et al., 2015), the results from both of these surfaces suggest that individuals experience lower resistance in these areas, which are typically perceived as suboptimal for amphibians that are highly susceptible to water loss (Spotila & Berman, 1976). Although these results seem counterintuitive, previous work in terrestrial salamanders suggests that individuals will move more quickly and directly through unfavorable areas in which they are physiologically stressed (Peterman, Connette, Semlitsch, & Eggert, 2014; Semlitsch et al., 2012). However, this observation could also be a consequence of pond placement as 19.57% of ponds are constructed on ridgetops and upper slopes and 57.30% of ponds are constructed on flat areas located on the ridgetops of FLW. As all species used in our study are dependent on ponds for breeding and the ponds act as stepping stones for dispersal, the fact that *A. annulatum* show lower resistance to movement on ridge tops could be an artifact of the pond configuration on our landscape. Additionally, *A. annulatum* have been observed moving through old field and pasture habitats toward breeding ponds (Briggler, Johnson, & Rambo, 2004) despite this habitat type leading to decreased survival in many species of *Ambystoma* likely due to increased desiccation risk and predator abundance (Rittenhouse & Semlitsch, 2006; Rothermel, 2004; Rothermel & Semlitsch, 2002) and higher resistance for gene flow than forested habitats (Crawford, Peterman, Kuhns, & Eggert, 2016; Greenwald et al., 2009).

For *A. opacum* and *N. v. louisianensis*, eastness emerged as the top model and northness emerged as the top model for *A. maculatum*. These surfaces approximate the temperature and soil moisture of a landscape as north‐ and east‐facing aspects are cooler and moister than south‐ and west‐facing aspects. Given that amphibians are prone to desiccation (Peterman, Locke, & Semlitsch, 2013; Spotila & Berman, 1976), a lower resistance of north and east facing aspects would indicate that soil moisture may be driving the increased genetic connectivity for individuals moving through these areas. For *A. opacum*, resistance was lower on western slopes, which are typically warmer and drier, than on slopes with easterly aspects. Similarly, *A. maculatum* resistance was higher on southerly aspects than on northerly aspects. The result for both species suggests that, like *A. annulatum*,* A. opacum,* and *A. maculatum* may exhibit compensatory movement through dry areas in the study landscape. In contrast, westerly aspects were estimated to have greater resistance for *N. v. louisianensis* than the typically moister easterly aspects. However, *N. v. louisianensis* overall *F’*_ST_ was extremely low and it exists as one genetic cluster across our study landscape. Thus, *N. v. louisianensis* are less prone to desiccation than the ambystomatids due to their granular skin (Gill, 1978), which may facilitate dispersal through a wider variety of habitat.

The support for a composite surface of TPI and distance from ravines explaining the genetic differentiation for *A. annulatum* and eastness describing genetic differentiation for *A. maculatum* extends previous work with *A. annulatum* and *A. maculatum* on this landscape. Peterman et al. (2015) found that no landscape surface described genetic differentiation demonstrably better than distance alone and our work expanding the number of ponds landscape surface described genetic differentiation demonstrably better than distance alone. Our study differed from that of Peterman et al. (2015) in that our *A. maculatum* samples were collected from a larger extent and we used TPI as a discrete variable with a 90 × 90 m cell size instead of using TPI as a continuous surface with a 30 × 30 m cell size. Although inference in “ResistanceGA” does not substantially change based on grid cell size (Peterman, 2014), our resistance surfaces likely encompass more heterogeneity in landscape features which can lead to different landscape genetic inferences (Short Bull et al., 2011).

In addition to landscape resistance predicting genetic differentiation, a likely explanation for the observed patterns of genetic diversity in our study is the effect of breeding phenology. We observed higher degrees of genetic differentiation and the presence of genetic clusters for the fall breeding species, *A. annulatum* and *A. opacum*, and a lower degree of genetic differentiation and a lack of genetic clustering in the spring breeding species, *A. maculatum* and *N. v. louisianensis*. In Missouri, *A. annulatum* and *A. opacum* breed and oviposit from September to early November and their larvae overwinter in the ponds before metamorphosing in late April to early June; thus, they require ponds that are continuously inundated and large enough to not freeze solid during that 6–9‐month period for successful reproduction (Anderson, Ousterhout, Peterman, Drake, & Semlitsch, 2015; Hocking et al., 2008; Urban, 2007). Both *A*. *maculatum* and *N. v. louisianensis* breed and oviposit in February and March and larvae metamorphose and disperse in a large pulse between early June and late July, although metamorphosis can continue into October, meaning that these two species can utilize more ephemeral ponds on the landscape as larvae can metamorphose in as few as 3 months (Gill, 1978; Hocking et al., 2008; Semlitsch & Anderson, 2016). Although metamorphosis is often prolonged, under stressful environmental conditions, such as pond drying, amphibian larvae are able to initiate metamorphosis more quickly if individuals are larger than the threshold size for metamorphosis (Semlitsch, 1987; Semlitsch & Wilbur, 1988).

Our observation of differential patterns of genetic structure between spring and fall breeding salamander species concurs with previous genetic studies of ambystomatid species that investigated patterns of genetic differentiations in species with different life histories. In Missouri, Peterman et al. (2015) found that the fall breeding *A. annulatum* had higher levels of genetic differentiation than the spring breeding *A. maculatum* on the same landscape. Similarly, Whiteley et al. (2014) observed that the fall breeding *A. opacum* has stronger genetic differentiation than *A. maculatum* among the same ponds in Massachusetts. From an evolutionary standpoint, the observed differences in dispersal distances and genetic structure between fall breeding and spring breeding species may be a result of the trade‐off between larval period length and size at metamorphosis (Petranka, 2007). Organisms with longer larval periods are able to reach a larger size at metamorphosis, a relationship that is directly linked to fitness and size at first reproduction (Scott, 1994), but they are able to utilize fewer breeding habitats in a given landscape (i.e., limited to more permanent ponds). Alternatively, the limited availability of suitable breeding habitat for fall breeding species compared to spring breeding species may have led to higher rates of philopatry in fall breeding species, as suggested by Peterman et al. (2015), because returning to successful breeding habitat may convey a fitness advantage, which may lead to decreased metapopulation connectivity (Petranka, 2007). This decreased connectivity leads to decreased gene flow in the metapopulation, as observed in our fall breeding species, which in turn lowers the potential for demographic rescue (Greenwald, 2010). The potential for demographic rescue is especially important for species, such as those used in this study, that have limited dispersal ability and boom or bust population cycles as the long‐term persistence and health of the species is reliant on migration and re‐colonization from other populations on the landscape in the event of local population crashes or bottlenecks (Greenwald, 2010).

Had our study only used one caudate species as a surrogate for all of the others, our inference would have differed as we would not have had the ability resolve the influence of breeding phenology on genetic differentiation in our system. Similar comparative landscape genetic studies support this idea that genetic inference can vary substantially, even among closely related and widely distributed species, when there are subtle differences in life history (Engler, Balkenhol, Filz, Habel, & Rödder, 2014; Kierepka et al., 2016). As such, we urge stake holders to make decisions using knowledge of multiple species on the landscape even if decisions are targeted toward a single taxon.

## CONFLICT OF INTEREST

None declared.
