# Supplementary material for: The influence of breeding phenology on the genetic structure of four pond‐breeding salamanders
Source: Ecol Evol. 2017 May 22;7(13):4670–81. doi: 10.1002/ece3.3060 (PMC5496555; doi:10.1002/ece3.3060)
Supplement: Supplementary file 1 [file ECE3-7-4670-s001.docx]

**SUPPLEMENTAL INFORMATION**

**Figure S1:** Maps depicting the spatial genetic clustering identified by BAPS. Results from this clustering analysis are largely concordant with STRUCTURE (Figure 3).

**Figure S2:** ‘ResistanceGA’ optimization surface transformation output for distance from ravine in *Ambystoma annulatum*. The inverse monomolecular transformation resulted in areas closer to the ravine having much greater cost than areas farther from the ravine.
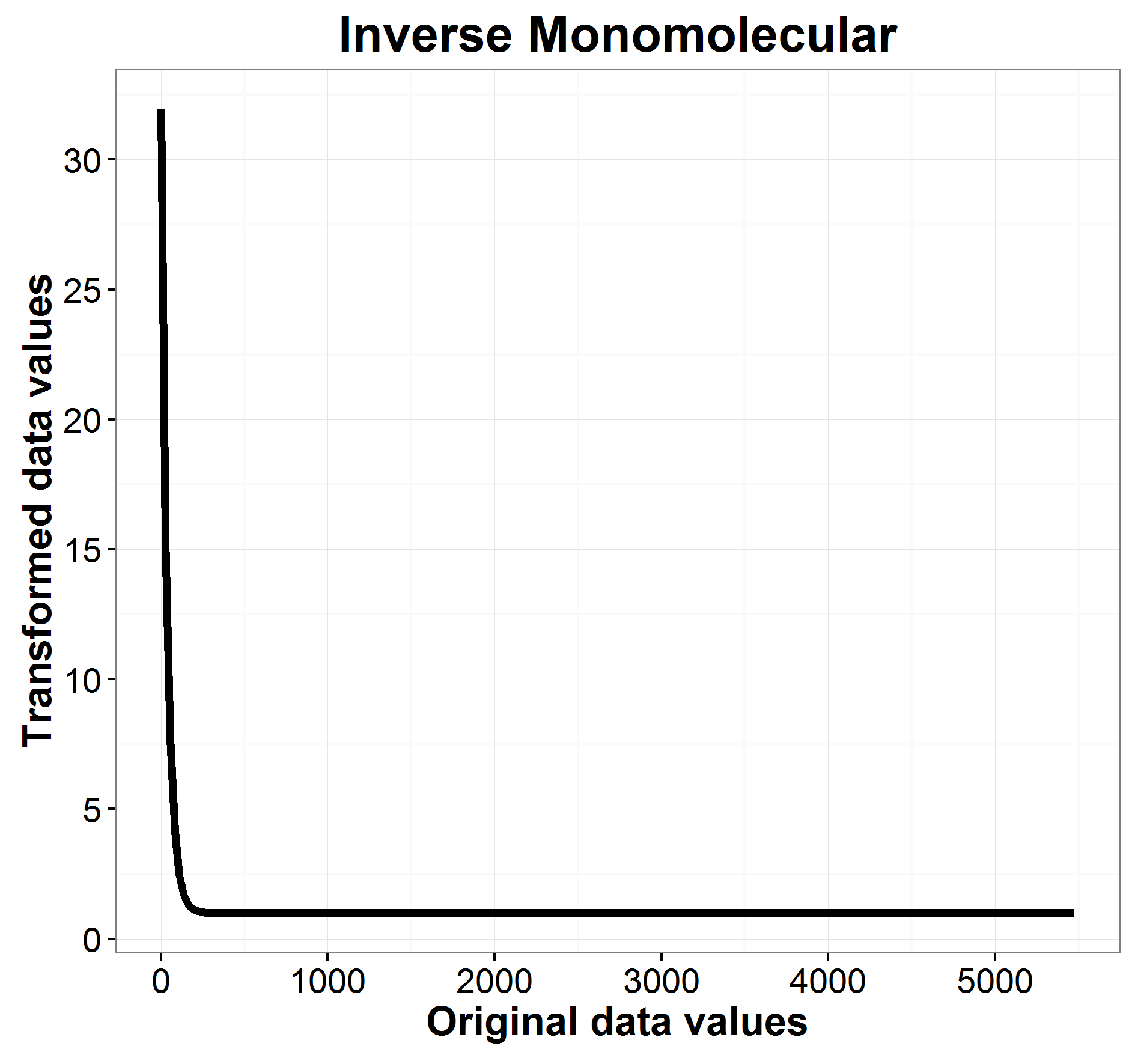


**Figure S3:** ‘ResistanceGA’ optimization surface transformation output for the best performing models in *Ambystoma opacum*: (A) eastness, (B) TWI, (C) distance from ravines, and (D) percent slope*.* Plot titles correspond to the transformation applied for optimizing the resistance curves in ‘ResistanceGA.’


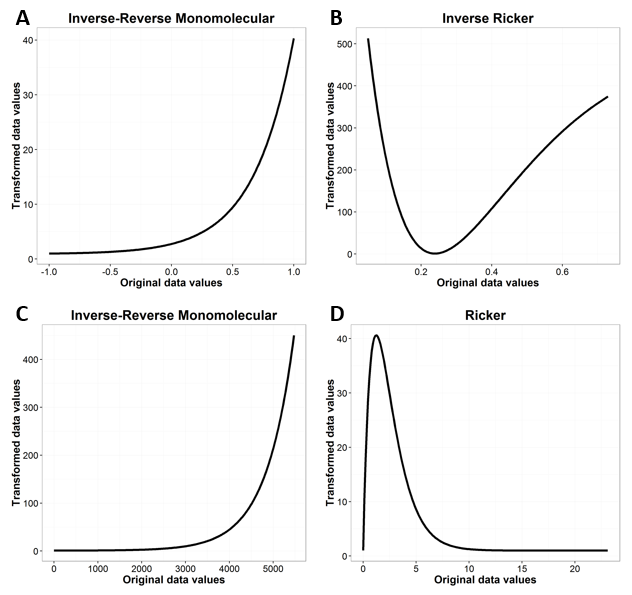


**Figure S4:** ‘ResistanceGA’ optimization surface transformation output for the best performing models in *Ambystoma maculatum:* (A) northness, (B) eastmess, and (C) percent slope*.*


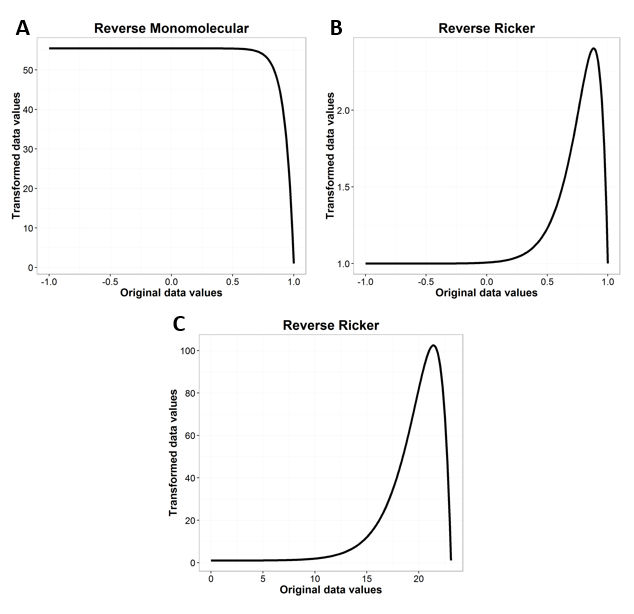


**Figure S5:** ‘ResistanceGA’ optimization surface transformation output for the best performing model, eastness, in *Notophthalmus viridescens louisianensis.* This transformation resulted in western aspects, which are typically warmer and drier, to have higher resistance than eastern aspects.


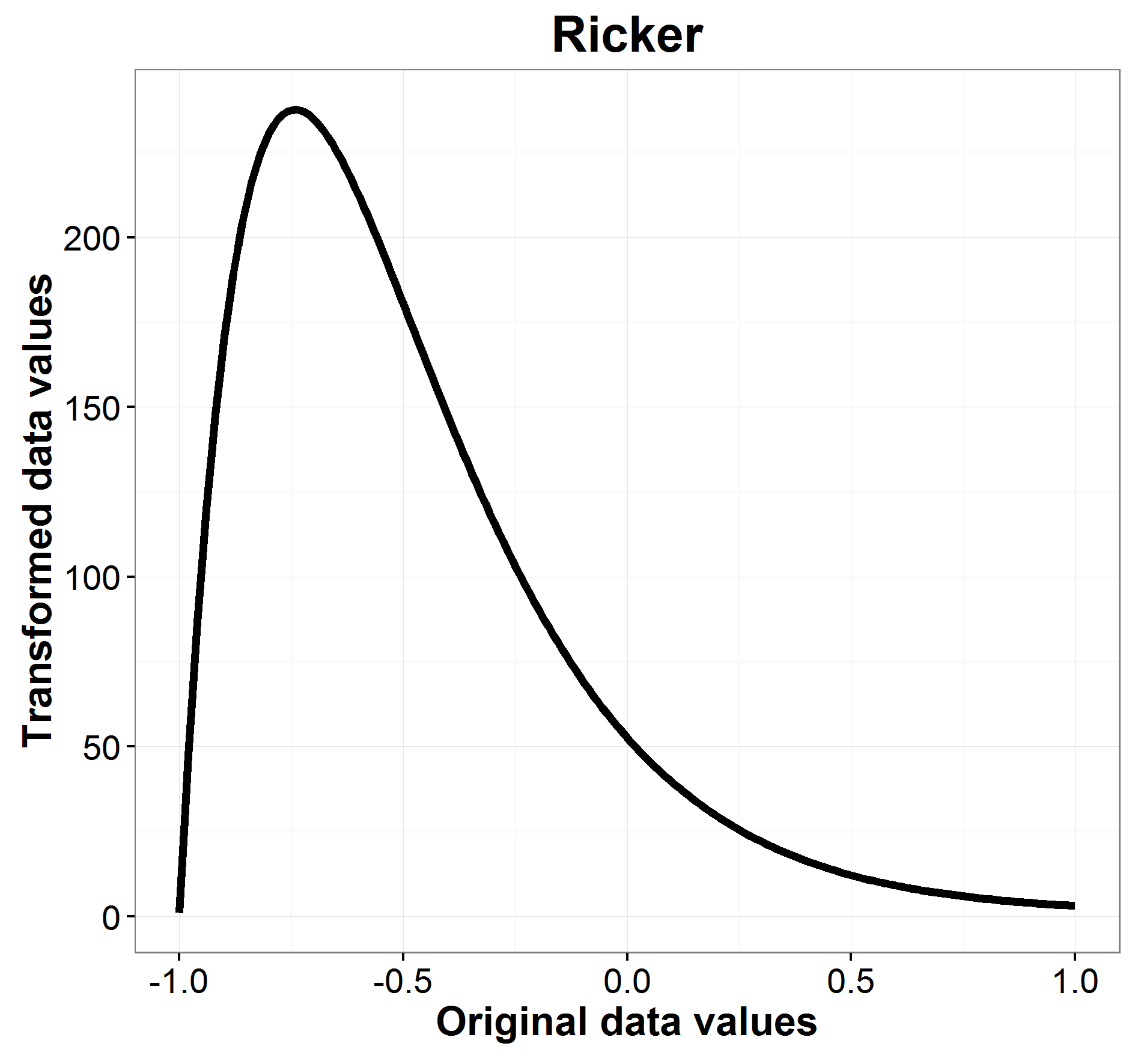


**Table S1:** Genetic diversity metrics for *Ambystoma annulatum* by (A) locus and (B) population.

A.

| **Locus** | **A** | **A_R_** | **Ho** | **He** | **F_IS_** |
| --- | --- | --- | --- | --- | --- |
| Aa-31 | 7 | 5.54 | 0.696 | 0.740 | 0.060 |
| Aa-311 | 16 | 7.77 | 0.873 | 0.825 | -0.058 |
| Aa-36 | 10 | 5.84 | 0.723 | 0.747 | 0.033 |
| Aa19 | 18 | 9.88 | 0.85 | 0.876 | 0.030 |
| Aa-21 | 10 | 5.89 | 0.744 | 0.758 | 0.020 |
| Aa-27 | 6 | 3.47 | 0.729 | 0.641 | -0.136 |
| Aa-86 | 3 | 2.44 | 0.409 | 0.404 | -0.011 |
| Aa-153 | 6 | 3.09 | 0.531 | 0.523 | -0.016 |
| Aa-84 | 4 | 2.75 | 0.554 | 0.561 | 0.013 |
| Aa-314 | 7 | 4.15 | 0.517 | 0.543 | 0.048 |
| Aa-20 | 5 | 3.64 | 0.520 | 0.599 | 0.132 |
| Aa-50 | 10 | 6.54 | 0.759 | 0.761 | 0.003 |
| Aa-25 | 9 | 6.06 | 0.823 | 0.783 | -0.052 |
| Aa-258 | 5 | 3.68 | 0.583 | 0.562 | -0.038 |
| Aa-85 | 6 | 5.60 | 0.828 | 0.780 | -0.062 |
| Aa-46 | 9 | 5.66 | 0.587 | 0.760 | 0.227 |
| Aa-39 | 5 | 4.28 | 0.622 | 0.651 | 0.045 |
| Aa-40 | 10 | 7.32 | 0.848 | 0.836 | -0.014 |
| Aa-312 | 9 | 5.61 | 0.753 | 0.748 | -0.006 |
| **Average** | **8.16** | **5.22** | **0.681** | **0.689** | **0.012** |
| **St. Dev.** | **3.83** | **1.85** | **0.138** | **0.127** | **0.077** |

A = total number of alleles per locus, A_E_ = the number of alleles in a population weighted for their frequencies; H_O_ = observed heterozygosity; H_E_ = expected heterozygosity; F_IS_ = inbreeding coefficient

B.

| **Pond** | **A** | **A_R_** | **H_O_** | **H_E_** | **F_IS_** | **F’_ST_** |
| --- | --- | --- | --- | --- | --- | --- |
| 0.002 | 5.737 | 3.764 | 0.631 | 0.684 | 0.078 | 0.145 |
| 120 | 4.947 | 3.545 | 0.700 | 0.720 | 0.029 | 0.188 |
| 127 | 5.053 | 3.296 | 0.677 | 0.690 | 0.019 | 0.159 |
| 152 | 4.895 | 3.589 | 0.666 | 0.688 | 0.033 | 0.159 |
| 213 | 5.053 | 3.673 | 0.702 | 0.705 | 0.004 | 0.167 |
| 216 | 6.053 | 4.043 | 0.720 | 0.737 | 0.023 | 0.099 |
| 238 | 5.421 | 3.696 | 0.705 | 0.686 | -0.028 | 0.143 |
| 242 | 6.421 | 4.300 | 0.741 | 0.733 | -0.012 | 0.090 |
| 245 | 4.474 | 3.345 | 0.738 | 0.698 | -0.057 | 0.113 |
| 246 | 5.474 | 4.095 | 0.729 | 0.728 | -0.001 | 0.132 |
| 247 | 5.842 | 3.991 | 0.690 | 0.714 | 0.034 | 0.110 |
| 302 | 5.842 | 3.545 | 0.662 | 0.662 | 0.000 | 0.153 |
| 311 | 4.632 | 3.260 | 0.688 | 0.669 | -0.030 | 0.159 |
| 314 | 4.684 | 3.436 | 0.737 | 0.690 | -0.068 | 0.129 |
| 321 | 4.579 | 3.001 | 0.629 | 0.623 | -0.009 | 0.219 |
| 331 | 6.158 | 3.821 | 0.708 | 0.706 | -0.004 | 0.126 |
| 339 | 3.684 | 2.605 | 0.619 | 0.605 | -0.023 | 0.228 |
| 355 | 4.947 | 3.497 | 0.690 | 0.679 | -0.017 | 0.163 |
| 380 | 6.526 | 3.960 | 0.723 | 0.709 | -0.019 | 0.100 |
| 400 | 6.053 | 3.600 | 0.595 | 0.676 | 0.119 | 0.167 |
| 408 | 4.211 | 3.081 | 0.644 | 0.667 | 0.036 | 0.302 |
| 414 | 5.579 | 3.751 | 0.663 | 0.711 | 0.067 | 0.103 |
| 66 | 5.263 | 3.465 | 0.642 | 0.686 | 0.064 | 0.129 |
| 71 | 5.842 | 3.851 | 0.690 | 0.711 | 0.030 | 0.118 |
| 8 | 5.789 | 3.657 | 0.699 | 0.690 | -0.013 | 0.135 |
| 82 | 4.316 | 2.973 | 0.631 | 0.652 | 0.032 | 0.198 |
| **Average** | **5.288** | **3.571** | **0.682** | **0.690** | **0.011** | **0.151** |
| **St. Dev.** | **0.732** | **0.386** | **0.031** | **0.031** | **0.042** | **0.047** |

A = number of alleles A_R_ = rarified allelic richness; H_O_ = observed heterozygosity; H_E_ = expected heterozygosity; F_IS_ = inbreeding coefficient; F’_ST_ = standardized measure of population differentiation (Meirmans, 2006)

**Table S2:** Genetic diversity statistics and multiplex information for *A. opacum* by (A) locus and (B) population.

A.

| **Locus** | **Label** | **Multiplex** | **A** | **A_R_** | **H_O_** | **H_E_** | **F_IS_** |
| --- | --- | --- | --- | --- | --- | --- | --- |
| AMOP-42 | FAM | 1 | 11 | 6.55 | 0.792 | 0.807 | 0.019 |
| AMOP-29 | VIC | 1 | 7 | 2.89 | 0.267 | 0.285 | 0.064 |
| AMOP-27 | NED | 1 | 13 | 6.16 | 0.751 | 0.786 | 0.045 |
| AMOP-18 | NED | 1 | 7 | 4.35 | 0.537 | 0.536 | -0.002 |
| Aa-20 | FAM | 2 | 2 | 1.82 | 0.165 | 0.262 | 0.371 |
| AMOP-31 | FAM | 2 | 16 | 5.46 | 0.395 | 0.839 | 0.529 |
| Aa-25 | VIC | 2 | 2 | 1.90 | 0.15 | 0.289 | 0.482 |
| AMOP-40 | VIC | 2 | 6 | 2.61 | 0.245 | 0.247 | 0.012 |
| AMOP-47 | NED | 2 | 9 | 2.59 | 0.377 | 0.650 | 0.420 |
| AMOP-33 | NED | 2 | 9 | 4.12 | 0.337 | 0.753 | 0.552 |
| Am-10 | PET | 2 | 15 | 5.22 | 0.823 | 0.827 | 0.005 |
| **Average** | - | - | **8.82** | **3.97** | **0.440** | **0.571** | **0.230** |
| **St. Dev.** | - | - | **4.67** | **1.71** | **0.249** | **0.253** | **0.239** |

Label = fluorescent dye used for each locus; Multiplex = multiplex panel that microsatellite primers were grouped into; A = number of alleles; A_R_ = rarified allelic richness; H_O_ = observed heterozygosity; H_E_ = expected heterozygosity; F_IS_ = inbreeding coefficient

B.

| **Pond** | **A** | **A_R_** | **H_O_** | **H_E_** | **F_IS_** | **F'_ST_** |
| --- | --- | --- | --- | --- | --- | --- |
| Demo | 5.182 | 3.092 | 0.402 | 0.596 | 0.325 | 0.083 |
| 103’s | 4.364 | 2.630 | 0.369 | 0.477 | 0.227 | 0.095 |
| 200’s | 5.727 | 3.535 | 0.519 | 0.608 | 0.146 | 0.010 |
| 274 | 5.545 | 3.166 | 0.525 | 0.630 | 0.168 | 0.024 |
| 356 | 3.909 | 2.983 | 0.458 | 0.584 | 0.215 | 0.020 |
| 36 | 4.182 | 2.695 | 0.438 | 0.558 | 0.215 | 0.075 |
| 387 | 4.636 | 3.188 | 0.405 | 0.592 | 0.316 | -0.001 |
| 419 | 4.273 | 2.646 | 0.403 | 0.525 | 0.233 | 0.110 |
| **Average** | **4.727** | **2.992** | **0.440** | **0.571** | **0.231** | **0.052** |
| **St. Dev.** | **0.675** | **0.319** | **0.057** | **0.050** | **0.063** | **0.043** |

Label = fluorescent dye used for each locus; A = number of alleles; A_R_ = rarified allelic richness; H_O_ = observed heterozygosity; H_E_ = expected heterozygosity; F_IS_ = inbreeding coefficient; F’_ST_ = standardized measure of population differentiation (Meirmans, 2006)

**Table S3:** Genetic diversity metrics for *A. maculatum* (A) by locus and (B) by population.

A.

| **Locus** | **A** | **A_R_** | **H_O_** | **H_E_** | **F’_IS_** |
| --- | --- | --- | --- | --- | --- |
| Am-56 | 13 | 2.84 | 0.541 | 0.688 | 0.213 |
| Am-34 | 10 | 6.00 | 0.633 | 0.791 | 0.201 |
| Am-62 | 4 | 2.88 | 0.36 | 0.413 | 0.129 |
| Am-4 | 14 | 6.86 | 0.788 | 0.808 | 0.025 |
| Am-39 | 5 | 5.67 | 0.619 | 0.690 | 0.102 |
| Am-9 | 12 | 4.34 | 0.723 | 0.729 | 0.009 |
| Am-21 | 8 | 5.72 | 0.695 | 0.678 | -0.025 |
| Am-10 | 14 | 6.99 | 0.652 | 0.742 | 0.121 |
| Am-29 | 8 | 3.95 | 0.47 | 0.517 | 0.092 |
| Am-43 | 3 | 2.79 | 0.417 | 0.398 | -0.046 |
| Am-33 | 5 | 2.62 | 0.195 | 0.189 | -0.034 |
| Am-13 | 4 | 2.35 | 0.175 | 0.171 | -0.024 |
| Am-30 | 7 | 5.02 | 0.716 | 0.724 | 0.011 |
| Am-38 | 4 | 3.38 | 0.557 | 0.546 | -0.022 |
| Am-3 | 15 | 10.10 | 0.9 | 0.877 | -0.027 |
| Am-55 | 6 | 3.50 | 0.589 | 0.581 | -0.015 |
| Am-7 | 9 | 6.32 | 0.809 | 0.762 | -0.061 |
| Am-37 | 6 | 4.42 | 0.543 | 0.533 | -0.020 |
| **Average** | **8.17** | **4.76** | **0.577** | **0.602** | **0.042** |
| **St. Dev.** | **3.85** | **1.97** | **0.192** | **0.197** | **0.083** |

B.

| **Pond** | **A** | **A_R_** | **H_O_** | **H_E_** | **F_IS_** | **F'_ST_** |
| --- | --- | --- | --- | --- | --- | --- |
| Demo | 5.611 | 3.205 | 0.593 | 0.618 | 0.039 | 0.059 |
| 0.002 | 4.667 | 3.104 | 0.616 | 0.637 | 0.033 | 0.052 |
| 107 | 5.333 | 3.525 | 0.594 | 0.637 | 0.067 | 0.087 |
| 129 | 5.500 | 3.461 | 0.605 | 0.644 | 0.060 | 0.091 |
| 152 | 4.500 | 2.929 | 0.556 | 0.577 | 0.038 | 0.067 |
| 159 | 5.222 | 2.858 | 0.521 | 0.568 | 0.084 | 0.070 |
| 199 | 4.778 | 2.920 | 0.563 | 0.564 | 0.000 | 0.061 |
| 200 | 5.500 | 3.126 | 0.581 | 0.597 | 0.027 | 0.053 |
| 249 | 5.444 | 3.113 | 0.569 | 0.577 | 0.015 | 0.043 |
| 274 | 5.000 | 2.993 | 0.581 | 0.607 | 0.044 | 0.069 |
| 282 | 5.278 | 2.925 | 0.552 | 0.580 | 0.048 | 0.057 |
| 290 | 5.278 | 3.358 | 0.622 | 0.614 | -0.012 | 0.087 |
| 306 | 5.167 | 3.073 | 0.593 | 0.614 | 0.034 | 0.072 |
| 36 | 5.611 | 3.229 | 0.597 | 0.631 | 0.053 | 0.046 |
| 387 | 4.722 | 2.739 | 0.554 | 0.575 | 0.037 | 0.070 |
| 79 | 5.222 | 2.919 | 0.506 | 0.575 | 0.120 | 0.062 |
| 8 | 5.389 | 3.099 | 0.602 | 0.618 | 0.027 | 0.052 |
| **Average** | **5.190** | **3.093** | **0.577** | **0.602** | **0.042** | **0.065** |
| **St. Dev.** | **0.331** | **0.207** | **0.031** | **0.026** | **0.030** | **0.014** |

A = number of alleles; A_R_ = rarified allelic richness; H_O_ = observed heterozygosity; H_E_ = expected heterozygosity; F_IS_ = inbreeding coefficient; F’_ST_ = standardized measure of population differentiation (Meirmans, 2006)

**Table S4:** Genetic diversity statistics and microsatellite primer information for *Notophthalmus viridescens louisianensis* by (A) locus and (B) population.

A.

| **Locus** | **Label** | **A** | **A_R_** | **H_O_** | **H_E_** | **F_IS_** |
| --- | --- | --- | --- | --- | --- | --- |
| Nvi-19 | PET | 3 | 2.23 | 0.436 | 0.478 | 0.087 |
| Nper-26 | NED | 12 | 5.88 | 0.168 | 0.823 | 0.796 |
| Nper-30 | FAM | 14 | 7.50 | 0.582 | 0.826 | 0.295 |
| Nvi-11 | PET | 8 | 5.84 | 0.809 | 0.817 | 0.009 |
| Tgr-06 | VIC | 17 | 8.62 | 0.813 | 0.886 | 0.083 |
| Nvi-24 | VIC | 18 | 5.20 | 0.331 | 0.786 | 0.579 |
| Nvi-3 | VIC | 20 | 9.30 | 0.851 | 0.884 | 0.038 |
| Nvi-2 | VIC | 5 | 2.66 | 0.349 | 0.57 | 0.387 |
| Nvi-7 | NED | 5 | 3.92 | 0.564 | 0.583 | 0.033 |
| **Average** | **-** | **11.33** | **5.63** | **0.545** | **0.739** | **0.263** |
| **St. Dev.** |  | **6.32** | **2.49** | **0.244** | **0.153** | **0.281** |

B.

| **Pond** | **A** | **A_E_** | **H_O_** | **H_E_** | **F_IS_** | **F’_ST_** |
| --- | --- | --- | --- | --- | --- | --- |
| 11 | 6.222 | 4.394 | 0.589 | 0.765 | 0.230 | -0.046 |
| 152 | 5.889 | 4.152 | 0.526 | 0.754 | 0.302 | -0.065 |
| 186 | 4.889 | 3.347 | 0.599 | 0.678 | 0.117 | -0.019 |
| 219 | 5.667 | 4.236 | 0.553 | 0.764 | 0.276 | -0.077 |
| 224 | 5.667 | 3.541 | 0.504 | 0.710 | 0.291 | -0.016 |
| 238 | 5.222 | 3.882 | 0.507 | 0.751 | 0.325 | -0.199 |
| 246 | 5.556 | 4.114 | 0.558 | 0.747 | 0.253 | -0.022 |
| 274 | 6.556 | 4.600 | 0.556 | 0.765 | 0.274 | -0.076 |
| 355 | 5.556 | 3.854 | 0.520 | 0.719 | 0.278 | -0.010 |
| 387 | 5.778 | 3.723 | 0.550 | 0.717 | 0.233 | -0.003 |
| 71 | 5.778 | 4.307 | 0.531 | 0.759 | 0.301 | -0.048 |
| **Average** | **5.707** | **4.014** | **0.545** | **0.739** | **0.262** | **-0.053** |
| **St. Dev.** | **6.556** | **4.600** | **0.599** | **0.765** | **0.325** | **-0.003** |

Label = fluorescent dye used for each locus; A = number of alleles; A_R_ = rarified allelic richness; H_O_ = observed heterozygosity; H_E_ = expected heterozygosity; F_IS_ = inbreeding coefficient; F’_ST_ = standardized measure of population differentiation (Meirmans, 2006)

**Table S5:** Combined genetic and geographic distance matrix for *A. annulatum*. Values below the diagonal are pairwise F’_ST_ values calculated in GenoDive. Values above the diagonal are pairwise geographic distances between ponds in kilometers.

|  | **0.002** | **120** | **127** | **152** | **213** | **216** | **238** | **242** | **245** | **246** | **247** | **302** | **311** | **314** | **321** | **331** |
| --- | --- | --- | --- | --- | --- | --- | --- | --- | --- | --- | --- | --- | --- | --- | --- | --- |
| **0.002** | - | 2.53 | 3.51 | 2.49 | 2.06 | 0.44 | 2.16 | 2.94 | 4.61 | 2.22 | 2.70 | 2.82 | 2.95 | 3.07 | 5.46 | 2.93 |
| **120** | 0.209 | - | 1.30 | 2.46 | 1.04 | 2.63 | 1.63 | 1.40 | 2.49 | 2.76 | 2.32 | 2.87 | 3.01 | 3.09 | 2.96 | 3.09 |
| **127** | 0.225 | 0.157 | - | 2.37 | 2.34 | 3.72 | 1.75 | 0.88 | 1.20 | 2.86 | 2.07 | 4.15 | 4.29 | 4.36 | 2.08 | 4.36 |
| **152** | 0.201 | 0.152 | 0.155 | - | 3.01 | 2.90 | 0.83 | 1.48 | 2.95 | 0.56 | 0.35 | 4.67 | 4.83 | 4.93 | 4.38 | 4.86 |
| **213** | 0.167 | 0.229 | 0.166 | 0.155 | - | 1.98 | 2.22 | 2.32 | 3.54 | 3.13 | 2.98 | 1.85 | 2.00 | 2.09 | 3.83 | 2.07 |
| **216** | 0.024 | 0.169 | 0.159 | 0.145 | 0.128 | - | 2.51 | 3.22 | 4.85 | 2.65 | 3.09 | 2.47 | 2.59 | 2.70 | 5.59 | 2.56 |
| **238** | 0.131 | 0.153 | 0.186 | 0.058 | 0.179 | 0.091 | - | 0.92 | 2.60 | 1.17 | 0.76 | 3.96 | 4.11 | 4.21 | 3.83 | 4.15 |
| **242** | 0.100 | 0.047 | 0.082 | 0.076 | 0.055 | 0.050 | 0.064 | - | 1.70 | 1.98 | 1.20 | 4.17 | 4.32 | 4.41 | 2.93 | 4.38 |
| **245** | 0.123 | 0.151 | 0.025 | 0.166 | 0.158 | 0.075 | 0.106 | 0.044 | - | 3.51 | 2.60 | 5.31 | 5.45 | 5.51 | 1.68 | 5.53 |
| **246** | 0.139 | 0.103 | 0.127 | -0.002 | 0.119 | 0.078 | 0.091 | 0.060 | 0.095 | - | 0.91 | 4.66 | 4.81 | 4.92 | 4.90 | 4.83 |
| **247** | 0.112 | 0.097 | 0.116 | 0.008 | 0.163 | 0.033 | 0.036 | 0.047 | 0.064 | -0.011 | - | 4.71 | 4.87 | 4.97 | 4.06 | 4.90 |
| **302** | 0.137 | 0.254 | 0.229 | 0.192 | 0.165 | 0.046 | 0.160 | 0.128 | 0.175 | 0.154 | 0.146 | - | 0.15 | 0.26 | 5.28 | 0.22 |
| **311** | 0.105 | 0.242 | 0.202 | 0.269 | 0.201 | 0.041 | 0.168 | 0.119 | 0.145 | 0.207 | 0.181 | 0.042 | - | 0.11 | 5.39 | 0.11 |
| **314** | 0.108 | 0.217 | 0.166 | 0.183 | 0.145 | 0.044 | 0.185 | 0.105 | 0.122 | 0.149 | 0.141 | 0.037 | 0.050 | - | 5.42 | 0.17 |
| **321** | 0.188 | 0.252 | 0.204 | 0.250 | 0.214 | 0.238 | 0.223 | 0.148 | 0.107 | 0.219 | 0.201 | 0.269 | 0.270 | 0.306 | - | 5.49 |
| **331** | 0.113 | 0.215 | 0.163 | 0.142 | 0.162 | 0.033 | 0.141 | 0.093 | 0.103 | 0.103 | 0.094 | 0.051 | 0.079 | 0.029 | 0.233 | - |
| **339** | 0.231 | 0.310 | 0.191 | 0.227 | 0.247 | 0.194 | 0.242 | 0.208 | 0.136 | 0.241 | 0.204 | 0.254 | 0.280 | 0.199 | 0.251 | 0.234 |
| **355** | 0.071 | 0.227 | 0.218 | 0.267 | 0.195 | 0.045 | 0.201 | 0.101 | 0.107 | 0.178 | 0.088 | 0.146 | 0.186 | 0.087 | 0.223 | 0.115 |
| **380** | 0.034 | 0.170 | 0.164 | 0.137 | 0.095 | 0.012 | 0.107 | 0.039 | 0.100 | 0.115 | 0.084 | 0.053 | 0.047 | 0.044 | 0.155 | 0.042 |
| **400** | 0.132 | 0.210 | 0.215 | 0.187 | 0.202 | 0.121 | 0.164 | 0.105 | 0.189 | 0.158 | 0.164 | 0.083 | 0.100 | 0.059 | 0.298 | 0.069 |
| **408** | 0.318 | 0.289 | 0.318 | 0.320 | 0.291 | 0.254 | 0.296 | 0.237 | 0.274 | 0.309 | 0.275 | 0.308 | 0.266 | 0.212 | 0.395 | 0.300 |
| **414** | 0.081 | 0.143 | 0.135 | 0.123 | 0.175 | 0.010 | 0.109 | 0.072 | 0.061 | 0.058 | 0.048 | 0.036 | 0.048 | 0.003 | 0.230 | 0.011 |
| **66** | 0.152 | 0.156 | 0.074 | 0.093 | 0.134 | 0.121 | 0.089 | 0.063 | 0.049 | 0.113 | 0.106 | 0.176 | 0.151 | 0.157 | 0.118 | 0.142 |
| **71** | 0.131 | 0.111 | 0.059 | 0.133 | 0.114 | 0.074 | 0.086 | 0.014 | 0.054 | 0.105 | 0.070 | 0.169 | 0.161 | 0.130 | 0.146 | 0.154 |
| **8** | 0.172 | 0.230 | 0.064 | 0.150 | 0.120 | 0.100 | 0.131 | 0.071 | 0.056 | 0.145 | 0.106 | 0.172 | 0.167 | 0.132 | 0.166 | 0.123 |
| **82** | 0.216 | 0.202 | 0.177 | 0.197 | 0.204 | 0.190 | 0.173 | 0.135 | 0.151 | 0.241 | 0.174 | 0.248 | 0.252 | 0.225 | 0.174 | 0.198 |

**Table S5 Continued:**

|  | **339** | **355** | **380** | **400** | **408** | **414** | **66** | **71** | **8** | **82** |
| --- | --- | --- | --- | --- | --- | --- | --- | --- | --- | --- |
| **0.002** | 5.55 | 0.55 | 1.64 | 3.46 | 2.44 | 2.82 | 4.29 | 3.52 | 4.14 | 5.82 |
| **120** | 3.56 | 3.01 | 2.25 | 2.38 | 0.50 | 2.78 | 2.05 | 1.56 | 2.48 | 4.04 |
| **127** | 2.26 | 3.89 | 3.54 | 3.48 | 1.76 | 4.05 | 0.79 | 0.41 | 1.32 | 2.75 |
| **152** | 3.61 | 2.53 | 3.60 | 4.67 | 2.83 | 4.63 | 2.88 | 2.08 | 2.15 | 3.67 |
| **213** | 4.60 | 2.60 | 1.25 | 1.68 | 0.62 | 1.77 | 3.09 | 2.59 | 3.50 | 5.08 |
| **216** | 5.83 | 0.84 | 1.32 | 3.22 | 2.46 | 2.48 | 4.50 | 3.77 | 4.45 | 6.13 |
| **238** | 3.45 | 2.38 | 2.95 | 3.86 | 2.00 | 3.90 | 2.41 | 1.58 | 2.00 | 3.67 |
| **242** | 2.62 | 3.23 | 3.35 | 3.78 | 1.88 | 4.10 | 1.49 | 0.66 | 1.25 | 2.93 |
| **245** | 1.07 | 4.93 | 4.74 | 4.55 | 2.94 | 5.22 | 0.49 | 1.08 | 0.93 | 1.63 |
| **246** | 4.17 | 2.15 | 3.53 | 4.81 | 3.06 | 4.63 | 3.41 | 2.60 | 2.71 | 4.21 |
| **247** | 3.26 | 2.80 | 3.68 | 4.61 | 2.73 | 4.66 | 2.55 | 1.76 | 1.80 | 3.33 |
| **302** | 6.38 | 3.31 | 1.19 | 1.31 | 2.38 | 0.11 | 4.84 | 4.42 | 5.35 | 6.89 |
| **311** | 6.52 | 3.43 | 1.33 | 1.36 | 2.53 | 0.23 | 4.98 | 4.57 | 5.49 | 7.04 |
| **314** | 6.58 | 3.55 | 1.44 | 1.34 | 2.60 | 0.31 | 5.04 | 4.64 | 5.57 | 7.10 |
| **321** | 2.12 | 5.89 | 5.07 | 4.20 | 3.22 | 5.17 | 1.53 | 2.30 | 2.59 | 2.81 |
| **331** | 6.60 | 3.40 | 1.32 | 1.47 | 2.60 | 0.32 | 5.06 | 4.64 | 5.56 | 7.11 |
| **339** | - | 5.83 | 5.79 | 5.59 | 4.01 | 6.29 | 1.55 | 2.08 | 1.47 | 0.70 |
| **355** | 0.220 | - | 2.15 | 4.01 | 2.96 | 3.32 | 4.65 | 3.85 | 4.38 | 6.04 |
| **380** | 0.210 | 0.060 | - | 1.96 | 1.87 | 1.18 | 4.31 | 3.73 | 4.59 | 6.23 |
| **400** | 0.317 | 0.150 | 0.051 | - | 1.91 | 1.21 | 4.05 | 3.84 | 4.79 | 6.17 |
| **408** | 0.401 | 0.326 | 0.257 | 0.315 | - | 2.29 | 2.48 | 2.05 | 2.98 | 4.51 |
| **414** | 0.243 | 0.092 | 0.026 | 0.022 | 0.324 | - | 4.74 | 4.34 | 5.26 | 6.80 |
| **66** | 0.193 | 0.238 | 0.088 | 0.196 | 0.265 | 0.115 | - | 0.83 | 1.15 | 2.12 |
| **71** | 0.211 | 0.149 | 0.103 | 0.164 | 0.297 | 0.073 | 0.036 | - | 0.95 | 2.49 |
| **8** | 0.145 | 0.142 | 0.112 | 0.213 | 0.288 | 0.136 | 0.063 | 0.058 | - | 1.69 |
| **82** | 0.107 | 0.231 | 0.180 | 0.284 | 0.409 | 0.199 | 0.128 | 0.138 | 0.120 | - |

**Table S6:** Combined genetic and geographic distance matrix for *A. opacum*. Values below the diagonal are pairwise G’_ST_ values calculated in the gdistance package for R. Values above the diagonal are pairwise geographic distances between ponds in kilometers.

|  | **Demo** | **103's** | **200's** | **274** | **356** | **36** | **387** | **419** |
| --- | --- | --- | --- | --- | --- | --- | --- | --- |
| **Demo** | - | 5.80 | 2.23 | 4.08 | 3.82 | 5.04 | 0.41 | 5.41 |
| **103s** | 0.207 | - | 6.57 | 8.30 | 7.86 | 3.57 | 6.20 | 0.50 |
| **200** | -0.022 | 0.114 | - | 6.08 | 5.89 | 4.46 | 2.07 | 6.31 |
| **274** | 0.050 | 0.125 | -0.032 | - | 0.45 | 8.82 | 4.07 | 7.81 |
| **356** | 0.055 | 0.037 | -0.012 | -0.097 | - | 8.46 | 3.85 | 7.37 |
| **36** | 0.119 | 0.110 | 0.025 | 0.051 | 0.071 | - | 5.28 | 3.66 |
| **387** | -0.046 | 0.083 | -0.110 | -0.028 | -0.068 | 0.051 | - | 5.81 |
| **419** | 0.215 | -0.014 | 0.106 | 0.101 | 0.153 | 0.100 | 0.111 | - |

**Table S7:** Combined genetic and geographic distance matrix for *A. maculatum*. Values below the diagonal are pairwise F’_ST_ values calculated in the GenoDive. Values above the diagonal are pairwise geographic distances between ponds in kilometers.

|  | **Demo** | **0.002** | **107** | **129** | **152** | **159** | **199** | **200** | **249** | **274** | **282** | **290** | **306** | **36** | **387** | **79** | **8** |
| --- | --- | --- | --- | --- | --- | --- | --- | --- | --- | --- | --- | --- | --- | --- | --- | --- | --- |
| **Demo** | **-** | 5.46 | 11.65 | 10.31 | 4.38 | 6.49 | 6.27 | 2.26 | 3.72 | 4.08 | 11.65 | 6.53 | 8.03 | 5.04 | 0.43 | 7.21 | 2.59 |
| **0.002** | -0.006 | **-** | 6.94 | 5.17 | 2.49 | 7.99 | 11.16 | 5.60 | 9.15 | 8.71 | 8.29 | 2.10 | 13.27 | 1.81 | 5.72 | 10.67 | 4.14 |
| **107** | 0.102 | 0.036 | **-** | 7.00 | 9.40 | 10.18 | 15.84 | 10.66 | 15.29 | 15.42 | 3.58 | 7.88 | 19.67 | 6.63 | 12.01 | 13.84 | 11.02 |
| **129** | 0.095 | 0.075 | 0.070 | **-** | 6.11 | 12.85 | 16.29 | 10.77 | 13.81 | 12.73 | 10.08 | 3.81 | 17.52 | 6.68 | 10.47 | 15.80 | 8.26 |
| **152** | 0.064 | 0.025 | 0.118 | 0.060 | **-** | 8.99 | 10.62 | 5.53 | 7.71 | 6.71 | 10.72 | 2.31 | 11.46 | 3.72 | 4.47 | 10.96 | 2.15 |
| **159** | 0.055 | 0.091 | 0.096 | 0.118 | 0.103 | **-** | 6.45 | 4.23 | 8.32 | 10.11 | 8.19 | 10.04 | 12.44 | 6.25 | 6.87 | 3.70 | 8.40 |
| **199** | 0.052 | 0.086 | 0.093 | 0.121 | 0.083 | 0.045 | **-** | 5.59 | 4.51 | 7.14 | 14.47 | 12.64 | 6.97 | 10.04 | 6.29 | 3.60 | 8.83 |
| **200** | 0.041 | 0.077 | 0.099 | 0.105 | 0.074 | 0.002 | 0.039 | **-** | 4.90 | 6.10 | 10.06 | 7.25 | 9.34 | 4.47 | 2.65 | 5.43 | 4.38 |
| **249** | 0.043 | 0.050 | 0.068 | 0.091 | 0.058 | 0.032 | 0.021 | 0.013 | **-** | 2.64 | 14.95 | 9.99 | 4.45 | 8.72 | 3.43 | 7.22 | 5.59 |
| **274** | 0.088 | 0.028 | 0.113 | 0.087 | 0.038 | 0.123 | 0.073 | 0.087 | 0.030 | **-** | 15.70 | 9.00 | 4.81 | 8.82 | 3.66 | 9.64 | 4.62 |
| **282** | 0.064 | 0.064 | 0.051 | 0.072 | 0.046 | 0.055 | 0.036 | 0.052 | 0.027 | 0.073 | **-** | 9.87 | 19.40 | 7.19 | 12.06 | 11.85 | 11.81 |
| **290** | 0.084 | 0.062 | 0.120 | 0.054 | 0.048 | 0.122 | 0.115 | 0.091 | 0.083 | 0.047 | 0.097 | **-** | 13.77 | 3.91 | 6.68 | 12.53 | 4.46 |
| **306** | 0.083 | 0.072 | 0.114 | 0.119 | 0.083 | 0.096 | 0.044 | 0.056 | 0.041 | 0.029 | 0.071 | 0.081 | **-** | 13.06 | 7.68 | 10.47 | 9.33 |
| **36** | 0.053 | 0.037 | 0.041 | 0.051 | 0.053 | 0.026 | 0.060 | 0.004 | 0.033 | 0.069 | 0.033 | 0.085 | 0.071 | **-** | 5.39 | 9.14 | 4.64 |
| **387** | 0.009 | 0.039 | 0.097 | 0.108 | 0.071 | 0.066 | 0.048 | 0.041 | 0.044 | 0.110 | 0.065 | 0.120 | 0.115 | 0.049 | **-** | 7.43 | 2.54 |
| **79** | 0.059 | 0.058 | 0.095 | 0.129 | 0.088 | 0.022 | -0.001 | 0.040 | 0.035 | 0.081 | 0.054 | 0.120 | 0.044 | 0.039 | 0.067 | **-** | 9.70 |
| **8** | 0.052 | 0.038 | 0.081 | 0.099 | 0.063 | 0.070 | 0.059 | 0.024 | 0.013 | 0.023 | 0.054 | 0.063 | 0.034 | 0.028 | 0.069 | 0.062 | **-** |

**Table S8:** Combined genetic and geographic distance matrix for *N. v. louisianensis*. Values below the diagonal are pairwise F’_ST_ and values above the diagonal are pairwise geographic distances between ponds in kilometers.

|  | **11** | **152** | **186** | **219** | **224** | **238** | **246** | **274** | **355** | **387** | **71** |
| --- | --- | --- | --- | --- | --- | --- | --- | --- | --- | --- | --- |
| **11** | - | 3.06 | 2.94 | 3.85 | 3.91 | 2.70 | 2.79 | 9.18 | 0.85 | 6.07 | 3.94 |
| **152** | -0.002 | - | 4.98 | 3.49 | 3.21 | 0.83 | 0.56 | 6.71 | 2.53 | 4.47 | 2.07 |
| **186** | -0.041 | 0.062 | - | 3.13 | 3.47 | 4.23 | 5.00 | 9.23 | 3.74 | 5.61 | 4.52 |
| **219** | -0.118 | -0.041 | -0.091 | - | 0.42 | 2.70 | 3.86 | 6.10 | 4.14 | 2.52 | 1.89 |
| **224** | -0.01 | -0.106 | 0.046 | 0.001 | - | 2.45 | 3.62 | 5.78 | 4.12 | 2.26 | 1.50 |
| **238** | -0.158 | -0.336 | -0.122 | -0.338 | -0.152 | - | 1.17 | 6.62 | 2.38 | 3.98 | 1.55 |
| **246** | -0.002 | -0.08 | 0.003 | 0.008 | 0.039 | -0.125 | - | 7.26 | 2.15 | 5.01 | 2.58 |
| **274** | -0.081 | -0.09 | -0.031 | -0.117 | -0.024 | -0.19 | -0.087 | - | 8.99 | 3.66 | 5.24 |
| **355** | -0.005 | 0.081 | -0.093 | -0.066 | 0.047 | -0.159 | 0.029 | -0.024 | - | 6.12 | 3.82 |
| **387** | -0.028 | -0.001 | 0.025 | 0.026 | 0.019 | -0.156 | 0.022 | -0.005 | 0.045 | - | 2.43 |
| **71** | -0.013 | -0.138 | 0.056 | -0.032 | -0.02 | -0.254 | -0.028 | -0.112 | 0.045 | 0.02 | - |
